# Experiences and challenges to cross-sectoral care reported by patients with low back pain. A qualitative interview study

**DOI:** 10.1186/s12913-020-4952-x

**Published:** 2020-02-06

**Authors:** Lisbeth Petersen, Regner Birkelund, Berit Schiøttz-Christensen

**Affiliations:** 1 Medical Research Unit, Spine Centre of Southern Denmark, Lillebaelt Hospital, Østre Hougvej 55, 5500 Middelfart, Denmark; 2 Health Services Research Unit, Lillebaelt Hospital, The Department of Regional Health Research, University of Southern Denmark, Beriderbakken 4, 7100 Vejle, Denmark; 3 The Department of Regional Health Research, University of Southern Denmark, Østre Hougvej 55, 5500 Middelfart, Denmark

**Keywords:** Patient experience, Person-centred health care, Cross-sectoral care, Continuity of care, Coherency, Course of treatment, Low back pain, Qualitative study

## Abstract

**Background:**

Cross-sectoral care comprises interdisciplinary and coordinated efforts for patients with complex care needs involving various competencies and professions across the primary health care sector, hospital sector, and municipal services. Cross-sectoral care can increase the effectiveness of rehabilitation programmes, but the treatment courses often lack coherence. Establishing successful treatment pathways requires a better understanding of the health care challenges faced by patients with low back pain. The aim of this study was to explore how patients with low back pain experience cross-sectoral care.

**Method:**

A qualitative interview study including 25 patients with low back pain. Patients were recruited in connection with their appointment at the Spine Centre of Southern Denmark. Recruitment stopped when the interviews no longer added new knowledge to the subject. The data were analysed using a systematic text condensation approach.

**Results:**

Patients with low back pain experienced cross-sectoral care to be fragmented, with episodes lacking collaboration, information, and acknowledgement of their problem. They desired recognition of having a serious back problem and of being more than the diagnosis itself. Patients found it hard to keep track of their course of treatment due to a perceived lack of organisational support and collaboration between professionals. The patients called for more information about the treatment plan and the reasons for further referral in order to better understand and manage their treatment.

**Conclusion:**

Patients’ experiences indicate a need for a stronger person-centred approach in cross-sectoral care, in which the individual’s experiences of living with low back pain are taken into account.

## Introduction

Low back pain is the number one cause of disability globally [1]. In 2015, the global point prevalence of activity-limiting low back pain was 73%, implying that 540 million people were affected at any one time [1]. At some point in their lives, 80% of the Danish population will experience low back pain, and for 75% of them, this will occur when they are 30–60 years old [2]. Each year, around 800,000 Danes experience low back pain; half of them are treated in the health care system, and around one-quarter of these are assessed at a Danish hospital. Low back pain can cause extensive functional impairment that negatively affects health, work and social conditions, and reduces quality of life.

Many patients with low back pain who experience functional impairment are affected physically, mentally, cognitively, and socially [3]. They need a multi-pronged effort involving various competencies and professions across the primary health care sector, hospital sector, and municipal services. Patients’ course of treatment for low back pain requires collaboration, both interdisciplinary and cross-sectoral, as patients are treated by professionals from various disciplines working in different sectors. Patients requiring long-term interdisciplinary and cross-sectoral efforts have a special need for a coherent and coordinated effort if the rehabilitation process is to succeed [4].

Research literature shows that interdisciplinary and cross-sectoral collaboration promotes the effect of rehabilitation in terms of fewer and shorter hospitalisations, fewer postoperative complications, less dependency on help, faster return to the labour market, reduced sick leave from work, and higher patient satisfaction [5–7]. In a literature study of continuity of care, Haggerty et al. furthermore showed that coherence in the course of treatment led to patients feeling safe and secure, which in turn increased their trust in the health care system and their motivation for active involvement [8]. In spite of this, there are still barriers to cross-sectoral courses of treatment in Denmark and other countries [3, 5, 8, 9].

During their course of treatment, more than half of patients with low back pain are in contact with various health care professionals in different sectors, and patients often end up circling the health care system with repeated assessments at primary or secondary health care facilities [10]. The combination of long treatment courses and lack of coherent cross-sectoral collaboration tends to maintain patients in a state where they feel constantly ill, making it hard for them to recover from their back pain and reduced quality of life [10]. Considerable economic and social gains could be made if patients with low back pain were offered a coherent course of treatment that enables them to return to work and a meaningful everyday life after rehabilitation.

The aim of this study was to explore how patients with low back pain experience cross-sectoral care. By understanding the obstacles they face, we hope to contribute to establishing a more coherent course of treatment.

Danish patients with low back pain are typically offered a course of treatment that stretches across several sectors. Most cases of low back pain are handled in the primary care sector by general practitioners (GPs), chiropractors, and physiotherapists. If a person experiences low back pain, he or she can choose to consult his or her GP or a chiropractor. The GP will examine the patient, and if the GP does not find signs of serious pathology (red flags), the patient will be referred to physiotherapy (if needed). If a person chooses to consult a chiropractor, the chiropractor performs the same examination as the GP, and if no red flags are identified, most patients are treated by the chiropractor in the clinic.

If the low back pain has not improved after 8–12 weeks of treatment in primary healthcare, the patient is referred to a hospital or for further diagnostics.

In the secondary sector (hospital or specialised Spine Centre), the diagnostics are performed in one day only and can be multidisciplinary if needed. Subsequently, there are several possible outcomes depending on the nature of the problem. One out of four patients is referred to rehabilitation at the municipal health service, which by some, is called the tertiary sector. In Denmark, a municipality is the smallest political and organisational unit. Municipal rehabilitation is a publicly paid service that extends over approximately 12 weeks performed by physiotherapists employed by the municipality. Municipal rehabilitation often takes place in local rehabilitation centres. In Denmark, multidisciplinary rehabilitation centres for patients with low back pain do not exist, which is why collaboration across the different sectors is crucial.

## Methods

### Design

The project was conducted as a qualitative interview study. An inductive approach was used, allowing patterns and themes to emerge from the interview data before deciding on the theoretical approach [11]. Data were interpreted and analysed according to Kirsti Malterud’s systematic text condensation strategy. The strategy represents a pragmatic approach to data analysis and comprises of four steps: 1) total impression – from chaos to themes; 2) identifying and sorting meaning units – from themes to codes; 3) condensation – from code to meaning; 4) synthesizing – from condensation to descriptions and concepts [12].

### Setting and participants

The study took place at the Spine Centre of Southern Denmark in collaboration with six municipalities. The target group comprised patients aged 18–60 years who were assessed and treated in both primary and secondary sectors for functional impairment caused by low back pain. Patients who had surgery were excluded from the study.

Data were collected through individual interviews with 25 patients with low back pain. In order to conduct interviews with a wide range of patients that represented the population, patients were randomly chosen from a list of patients scheduled for assessment of low back pain at the medical department at the Spine Centre. They were contacted by phone a few days prior to their appointment at the Spine Centre and asked to participate in an interview in connection with their assessment at the Spine Centre. The interviews took place in May and June 2016 and lasted about half an hour. There was an equal division of women and men.

### Qualitative interviews

The interviews focused on the patients’ experiences during the course of their treatment. The patients were asked about challenges they had experienced and what aspects of their treatment had worked and had not worked.

The interviews were based on a semi-structured interview guide with the following topics.

- Experiences with the course of treatment
  - ○ Can you please tell me about your experiences as a back pain patient?
  - ○ How did you experience your course of treatment? (meetings with your GP, other professionals or the health care system as a whole)
  - ○ Good and bad experiences?
  - ○ Could anything have been different?
- Everyday life
  - ○ How does your low back pain affect your everyday life (work, education, family, leisure)?
  - ○ In your experience, what is the hardest part of having low back pain?
- Involvement
  - ○ How was your experience of being involved in your course of treatment?
  - ○ Did you receive the necessary information and knowledge you needed regarding your treatment?
- Needs
  - ○ Which challenges did you face throughout your course of treatment?
  - ○ What do you feel is needed for you to have a better life dealing with your low back pain?
- Where do you go to find answers to questions regarding your situation?
- What is your most important experience as a patient throughout your course of treatment?
- Do you have any advice or suggestions for improvement?

The interviews were conducted by two researchers. One researcher interviewed the informant and ensured that all aspects of the interview guide were discussed. The other researcher observed and listened to the interview while taking additional notes. This gave the observer time to reflect on statements from the informant and to ask in-depth questions that the interviewer might have missed when focusing on the flow of the interview. Both researchers have several years of experience conducting and analysing qualitative interview data.

### Ethics

The project was registered at the Danish Data Protection Agency with registration number 18/27313, and permission was granted by the management of the Spine Centre. The patients were informed by providing oral information, and written consent was obtained prior to all interviews. The patients were informed that they could withdraw their consent at any point during the project without consequences for their treatment. Data were depersonalised to ensure anonymity. According to Danish Act on Research Ethics Section 2 interviews is not required for conducting interviews as long as the data are anonymised.

### Data analysis

The interviews were recorded and transcribed into text, and the program Nvivo was used to structure and thematise the interview data. The data were interpreted and analysed according to Kirsti Malterud’s systematic text condensation strategy [12]. This method offered an overview and helped to structure the interview data. Two researchers collaborated in the meaning-condensation and analysis. The analytic process focused on the meanings, patterns, and important characteristics of the patients’ perceptions of their course of treatment [12]. The interview data were divided into themes from the patterns generated by re-reading the data. The essential themes were then linked together in descriptive statements. Thus, the method involved the condensation of expressed meanings in increasing significance regarding experiences and challenges for coherent cross-sectoral care for patients with low back pain.

## Results

The results are an interpretation of the main points from the patients’ thoughts and experiences. Three main themes emerged from the interview data: (1) lack of organisational support and collaboration, (2) lack of information, and (3) lack of acknowledgement.

### Lack of organisational support and collaboration

Reading through the interview data, it became clear that a course of treatment for patients with low back pain is not linear but rather a mix of assessments and treatments in the primary care sector, the Spine Centre, and the municipality. Patients with low back pain moved between these different health care facilities in what the patients called random order with no structure or explanation of what would come next and why. Patients experienced that they used a lot of time and energy working out their possibilities and rights in relation to aspects such as diagnostic imaging, assessments, treatment, and sick leave. For that reason, many patients found it hard to keep track of their course of treatment throughout the months or years of assessments and treatments. Most patients expressed a need for a co-ordinator who was responsible for maintaining an overview and connecting the strings between the assessments and treatments in the various health facilities. They also expressed a need for someone to regularly follow up on their situation and to help them navigate and make sense of their course of treatment.

Patients’ perceptions of their course of treatment seemed to depend on the role undertaken by the general practitioner (GP). When patients talked about their course of treatment, they emphasised that the GP played an important role. Some patients stated that their course of treatment had been successful and that their GP had initiated different steps and had followed up afterwards. Other patients said they lacked a plan and felt they were passed around the health care system without a concrete goal or someone to take responsibility for the course of treatment. One patient stated that she felt *“thrown around the system”* and for long periods of time was *“left to fend for herself”.*

Our data revealed a connection between coordination and patient satisfaction. Patients who stated that their GP was successfully filling the role as a co-ordinator generally expressed more satisfaction with their course of treatment than patients who felt their GP was not taking responsibility for following up and keeping track of their course of treatment.

Several patients had experienced lack of coordination and collaboration between health professionals as they moved between sectors. Lack of collaboration was experienced in different ways by the patients. Some stated that they sometimes felt that professionals from different sectors were waging war on each other and showed dislike or disrespect for each other in an unpleasant way.

The most common frustration expressed by patients was poor collaboration between the municipal jobcentre and the health care system. Some patients said that they often felt that professionals had different agendas or that they did not agree on the plan or the goal. This made the patients feel that the professionals were working in different directions. One patient expressed:

*“According to the regulations, I was supposed to meet the rehabilitation team during the first four weeks of my sick leave. Seven months passed, and nothing happened. Then, I met with the rehabilitation team, who told me that my work ability must be clarified before they [could] decide what to do with me. So, what have I been doing for the last seven months? Nothing. So, now I am trying to clarify my work situation and how much I can handle despite my back pain but … the municipality is stuck with this task and … I don’t know … . It could be nice to experience a bit more collaboration between the Spine Centre and the municipality. The professionals at the Spine Centre are the experts, but the social workers are not really listening … . It’s like they are at war sometimes”.*

Patients, thus put into words how they experienced a lack of coordination and that poor communication led to misunderstandings and increased waiting time. Several patients said they found themselves caught between the health care system and the municipal jobcentre, and the patient in the above statement shared this perception when he experienced the municipality to be passive and unable to take responsibility or to cooperate with the Spine Centre.

Another challenge in relation to lack of collaboration was the experience of having to start all over again, explaining their health situation, when seeing a new health care professional. One patient explained:

*“First, I see the physiotherapist where I have to explain everything I’ve been through, and then I return to the doctor and I have to explain it all again. There is no coherence. The professionals need to take a closer look at how many places you have been, how long have you had back pain, and what other clinicians had found, and then they should ‘check’ this off so they can say, ‘This has already been tried; now, let’s try something else’”.*

This statement reflects the patients’ experiences that the knowledge generated by the first professional did not appear to transfer to the next professional they met. Our data show that visible collaboration between professionals can increase patient satisfaction, as illustrated in the following statement:

*“The different clinicians had such a good communication. The clinician at the second hospital got all the information he needed from the clinician at the first hospital. It was like ... the clinician at the first hospital said; “I can’t do more for you at this point, but I know that this person at the other hospital can, so I will refer you to him, and he’ll take over exactly from here onwards”.*

This is an example of an experiences where the clinicians collaborated in a respectful way. The first clinician was honest when his examination showed that the patient’s condition fell outside his area of expertise, and he ensured that all the data were passed on when he referred the patient to a colleague at another hospital.

### Lack of information

Several patients said that lack of information was a challenge during their course of treatment. The patients talked about two types of information. The first concerned knowledge about low back pain and diagnostic and treatment opportunities, including information about physical symptoms and the anatomy of the back that could help the patients understand their pain. The patients wanted this kind of information to be communicated in understandable Danish instead of only Latin phrases as in their health records.

The second type of information was a visual image of the care plan with explanation of the various steps during the course of treatment. Being uncertain of the next treatment step was a concern expressed by many patients. When we asked the patients to explain to us the order of the assessments and treatments they had received, they rarely provided a linear timeline of experiences. On the contrary, they often stated that they felt they were running in circles or that their treatment seemed random and coincidental. Some patients questioned the treatment procedures with statements like: *“Why do I need to consult my GP when my medicine has already been cleared with the nurse at the Spine Centre?”* or *“Why do I need to go see a physiotherapist for three months instead of being directly referred to the Spine Centre?”* These patients wanted an explanation for why certain steps were initiated and how they should react when steps were completed or did not have the desired effect.

In addition to not knowing the care plan, several patients expressed frustration that they were given conflicting information by health professionals in different settings. Some patients had received varying advice on how to reduce low back pain. One patient said:

*“I felt like I was floating around, and I had to figure out, by myself, if I should see a physiotherapist or a chiropractor, should I go home and lay down or should I exercise? Should I put ice on it or should I use heat? Is it okay if it hurts when I exercise? There were so many different people telling me different things. I have experienced a physiotherapist saying that you should not do anything, you should take care of yourself, and then I saw another physiotherapist who said that I should exercise hard. And I know that I have to make my own decisions and it’s my responsibility, but it’s just so confusing”.*

The patients expressed a need for information that is concrete and specific. Contradictory or vague information about what to do or where to go next appears to challenge coherence as it confuses the patients and makes it harder for them to understand their course of treatment.

### Lack of acknowledgement

The communication in the physical meeting between a patient and a health care professional was of great importance for the patients’ experiences of their course of treatment.

Many patients stated that they had to insist, for a long time, that they had low back pain before the GP actually listened and acted. Some patients said that it was hard for them to get the GP to acknowledge their low back pain as a substantial issue, while others found that their GP did not allocate enough time in the consultation to listen properly to them.

One of the patients felt that she had been *“wasting her breath”,* and another said that *“it can take a long time to be taken seriously when having back pain”*. She had received a standard answer from her GP: *“Yes, your back hurts – do some exercise”.* One patient who was particularly frustrated with his GP was asked if his GP listened to him. He answered:

*“No, he most certainly does not. He does not even know what the matter is with me. My GP, he is so … he sometimes makes me so angry. Because he … . You get to say two words before he takes over the conversation, and he just bla bla bla, you get this medication. Was there anything else? I can’t get myself heard. I mean, I don’t see my GP every other day, so I do have a reason for seeing him. And when I finally see him, I expect to be listened to and be taken seriously, but the GP just rushes through the consultation”.*

The experience of not being listened to or taken seriously made patients feel that they have to be strong and insisting before anything is done. An important issue that patients also mentioned in this context was the time spent struggling to be acknowledged whilst not getting any treatment, thereby prolonging the course of treatment.

The patients who experienced their meeting with the health care professionals as positive described a feeling of being seen and met as an equal human being and not just as a patient. One patient who was very satisfied with her course of treatment at a health care facility said that she immediately felt that she had come to *“the right place”.* She liked the *“professionalism and the empathy shown by the health care professionals”*. Asked what she meant by professional and empathetic, she answered:

*“They were prepared. I was expected. It wasn’t like they needed to read my health records after I entered the room, and my MRI scan was already on the screen. It was me they were talking about, you know. And there was kindness and eye contact. There was a sense of warmth and human understanding that I was a person in pain that they could help”.*

Seeing the patient is not only to make eye contact, but as this statement shows, it is also being prepared and having read the health records before the patient enters the room.

## Discussion

Based on our results, the following discussion focuses on the patients’ need for being acknowledged for their situation throughout their course of treatment. Patients want to be recognised as having a serious back problem as well as recognised as a human being who is living and experiencing all aspects of life and is, therefore, much more than just a diagnosis. These results are in line with the newest literature on the subject [3, 5, 8, 9, 13, 14]. Our findings will be discussed through published research as well as theoretical perspectives on sense of coherence by Aaron Antonovsky [15] and person-centred care by Angela Coulter [13].

In a systematic review of illness experiences of patients with low back pain, Damsgaard et al. showed that the meeting between patient and professionals is a recurrent theme [9]. Patients experience that they are not taken seriously and that professionals over-emphasise symptoms and fail to listen to the patients, dismissing their daily experiences of living with low back pain. The result is that patients experience a loss of self-perception and social position while waiting for a diagnosis [9]. This constant battle to get health professionals to acknowledge their low back pain can reduce the patients’ belief in themselves and their experiences, leading to dissatisfaction with the way they are met by the health care system. It can also result in patients becoming dependent on the health care system to take responsibility for the course of treatment.

Damsgaard et al. argue that health professionals need to pay attention to the patient’s narratives and lifeworld in order to acknowledge the patient as a human being [9]. Lifeworld-led health care is an approach that provides deeper insight into patients’ experiences by understanding how the individual person lives and functions in the world. Patients’ narratives include their perception of their situation, their experiences of pain, and level of function as well as their thoughts on preferences and what is important to them in everyday life. The patient’s own view of the medical history and the course of low back pain is also part of these narratives.

The statements from the patients about not being listened to indicate that the patients do not experience their narratives as considered. As a result, it becomes the professional’s role to decide what is important to address instead of what patients consider important.

The patients’ experiences about not being viewed and acknowledged as human beings, with attention paid to their own experiences, lifeworld, values, and preferences, do not fit into the paradigm of person-centred health care that has found favour in recent years. Political visions in Denmark describe a health care system that works towards a culture of patient involvement where decisions and treatment are based on the patient’s own preferences [16]. In her book *Engaging Patients in Healthcare,* social scientist Angela Coulter sums up the essence of person-centred care as: “No decisions about me without me” [13]. According to Coulter, person-centred health care is about ensuring that care delivery is responsive to the patient’s physical, emotional, and social needs and puts the patient’s lifeworld at the centre of attention [13]. Wang et al. showed a clear relationship between patient-centred care coordination and patient satisfaction among patients with diabetes [5].

As shown in our results, even a small action, such as having read the health records before the patient enters the room, signals that the professionals have considered the patient’s medical history and are informed of the patient’s situation. The patient felt that by being prepared, the professionals took her seriously and made her feel understood and that they saw her as a human being they could help.

However, several patients expressed that they did not feel they were taken seriously in the meeting with the professionals, and they expressed dissatisfaction with GPs who were too busy to actually listen and acknowledge the patients’ low back pain. This suggests that these patients currently do not experience the health care system to be person-centred. Patients’ experiences of professionals giving opposing explanations and not having a consensus for best practice indicate a higher focus on medical practice at the expense of patients’ preferences.

A report on Danish patients’ perception of coherence in their course of treatment showed that cross-sectoral cooperation does not automatically define a coherent course of treatment for patients [14]. As in our study, the report showed that patients tend to think in episodes and not in a linear course of treatment; furthermore, that it is not always important that all standard regulations are respected as long as assessments and treatments are experienced as coherent. Coherence can be achieved by factors such as repeatedly meeting the same doctors and other staff, attention to details, and professionals articulating and taking responsibility for changes or mistakes [14]. Our results support these findings. The patients valued the professional being honest about their own shortcomings as long as the professional took responsibility and coordinated the next step by ensuring that the appropriate information was shared with other professionals while at the same time informing the patient of what is going on and why.

It appeared that patients viewed collaboration as an active part of knowledge sharing, where professionals from different sectors should communicate with each other to agree on the different aspects of a patient’s course of treatment. These agreements are needed to ensure correct treatment and adequate progress towards a common goal.

Our results revealed that much of the information given to the patients was neither precise nor consistent, and this made it hard for patients to understand the plan for their course of treatment. The patients that did receive accurate information, especially from their GP, were more satisfied with their course of treatment, whereas inaccurate information and failure to explain the next step led to patients feeling they were running in circles or were wasting time on what seemed to be random treatment. On the contrary, the lack of information transfer from one sector to another led patients to feel they were wasting time repeating themselves over and over again due to lost knowledge about what had already been assessed or tested.

### Challenges to cross-sectoral care

Cross-sectoral collaboration should serve to assure patients that they are being cared for by the appropriate specialists during their course of treatment, and such collaboration, therefore, calls for dialogue and mutual understanding, commitment, and agreement between professionals. But, as discussed, it seems that a coherent course of treatment does not solely depend on the different units working together. It is equally important that patients experience progress in their course of treatment, that they are informed of the steps of their trajectory, that professionals take responsibility, and that professionals acknowledge the patients’ experiences of their own situation and take them seriously.

A coherent course of treatment is thus one that pays attention to the person having a low back pain problem and does not focus solely on diagnosing the cause of the low back pain. Ensuring adequate information, coordination, and collaboration requires other values than finding and treating a biomedical diagnosis. Coherence seems to be found in the personal relationship between patient and professional where the dialogue is accompanied by acknowledgement, respect, transparency, and information that support the patient’s understanding of the low back pain situation and attend to the individual’s experiences of living with low back pain in everyday life.

Our study results are in line with the existing literature on cross-sectoral care. According to Antonovsky’s theory of sense of coherence, the ability to master challenges in everyday life is essential for a person’s health and depends on the individual’s sense of coherence, where life is perceived as understandable, manageable, and meaningful [15]. In Antonovsky’s perspective, patients must be able to understand their low back pain as well as their course of treatment and they need resources such as information, knowledge, and acknowledgement from their surroundings to allow them to manage their course of treatment. Lastly, the course of treatment should make sense for the patients to invest time and resources in it.

According to Hjortbak et al., coping in relation to a sense of coherence in the course of treatment can be improved through patients developing an understanding their own situation, experiencing a professionally competent and coordinated effort characterised by thorough communication and continuity, and having opportunities for relevant support [3]. Our study explored the elements of communication and continuity that patients with low back pain experience as important for coherency in their course of treatment.

A meta-summary on continuity of care for patients with a variety of conditions by Haggerty et al. [8] showed that coordinated care can be achieved by professionals sharing the information that is given to the patient and supporting an active patient role in giving and receiving information, monitoring, and self-management. Having a single, trusted clinician who helped them navigate the system and treated the patient as a partner was important to the experience of continuity as it helped patients to know what to expect, and the contingency plans provided security for the patients [8].

In spite of much knowledge on how to achieve continuity of care, cross-sectoral care in Denmark and other countries faces several challenges. Barriers to coherent courses of treatment can be lack of patient involvement, opposing explanatory models, incomplete information, standardisation, lack of a co-ordinator, unclear division of roles, and lack of collaboration and communication across sectors and between different groups of health professionals [5, 14, 17–19]. According to Haggerty et al., these factors create patient distrust of the clinician’s expertise and competencies and create doubt about which advice to follow [8].

Our study has identified the same challenges as other studies on coherent cross-sectoral care. This indicates that challenges to cross-sectoral care are not dependent on the patient’s condition but are influenced by the approach to patients as well as how cross-sectoral treatments are organised.

Most studies on cross-sectoral care for patients with a chronic condition such as low back pain focus on practice in relation to hospitalisation. This is partly because patients experience hospitalisation as the most challenging aspect of their course of treatment [14]. Our study population was mainly treated with ambulatory care, which differs from the typical cross-sectoral care described in the literature. We did, however, find that patients in ambulatory courses of treatment experienced the same challenges as described in relation to hospitalisation. It should be noted, therefore, that important transitions from one sector to the next occur in other forms of care besides those that include hospitalisation.

### Strengths and limitations of the study

The qualitative interviews provided rich material on patients’ experiences with their course of treatment. However, it might have provided more relevant knowledge to interview patients with low back pain after they ended their municipal rehabilitation programme. This would have provided a more varied description of patients’ experiences with their course of treatment from beginning to end. On the other hand, most of the interviewed patients had been dealing with low back pain for several months or even years and thus had experience with various assessments and treatments in primary health care, the Spine Centre, and municipal services.

It was considered important to interview a wide range of patients with various experiences with low back pain which is why the method of randomly selecting patients to participate was chosen. It should be noted that the results are not generalisable to all patients with low back pain, since only a selection of the patient group was interviewed.

## Conclusion

Patients’ experiences indicate a need for a stronger person-centred approach in cross-sectoral care in which the individual’s experiences of living with low back pain are taken into account.

Patients with low back pain, in this study, indicated that they were usually met by a biomedical view of pain and that their own experiences, lifeworld, and preferences were neglected in favour of a medical diagnosis. They experienced cross-sectoral care as being fragmented and lacking collaboration, information, and acknowledgement of their low back pain situation. The support and interventions provided as part of the cross-sectoral care for patients with low back pain would benefit from addressing the challenges experienced by patients.

Based on our findings, we suggest that focus should be on the large group of people with low back pain as they possibly represent patients with higher risk of developing chronic pain accompanied by worse health, increased health care consumption, and lower probabilities of returning to work.

## Data Availability

The datasets generated and/or analysed during the current study are available from the corresponding author on reasonable request.

## Abbreviation

GP: General practitioner
